# Association of intestinal pathogens with faecal markers of environmental enteric dysfunction among slum‐dwelling children in the first 2 years of life in Bangladesh

**DOI:** 10.1111/tmi.13141

**Published:** 2018-09-10

**Authors:** Shah Mohammad Fahim, Subhasish Das, Md. Amran Gazi, Mustafa Mahfuz, Tahmeed Ahmed

**Affiliations:** ^1^ Nutrition and Clinical Services Division icddr,b Dhaka Bangladesh

**Keywords:** Environmental enteric dysfunction, intestinal pathogens, giardiasis, trichuriasis, Bangladesh, dysfonction environnementale entérique, pathogènes intestinaux, giardiase, trichocéphalose, Bangladesh

## Abstract

**Objective:**

Environmental Enteric Dysfunction (EED) can be assessed by faecal biomarkers such as Myeloperoxidase (MPO), Neopterin (NEO) and Alpha‐1 anti‐trypsin (AAT). We aimed to test the association of intestinal pathogens with faecal markers of EED among slum‐dwelling children in first 2 years of life.

**Methods:**

The MAL‐ED birth cohort data of Bangladesh site were used to conduct this analysis. Multivariable analyses using Generalized Estimating Equations (GEE) were performed to test the association between intestinal pathogens and faecal markers of EED.

**Results:**

Giardiasis, ascariasis and trichuriasis were the most frequent parasitic infections and *Campylobacter* spp., Enteroaggregative *Escherichia coli* (EAEC) and Enterotoxigenic *Escherichia coli* (ETEC) were the common bacterial pathogens observed in stool samples of the children. Overall, 71%, 97% and 58% of stool samples were above values considered normal in non‐tropical settings for MPO, NEO and AAT respectively. Giardiasis was found to be significantly associated with MPO (Coefficient = 0.55; 95% CI = 0.15, 0.95; *P*‐value = 0.008) and AAT concentrations (Coefficient = 0.34; 95% CI = 0.04, 0.63; *P*‐value = 0.03). A significant association was found between trichuriasis and NEO (Coefficient = 0.90; 95% CI = 0.19, 1.61; *P*‐value = 0.01). Trichuriasis (Coefficient = 1.71; 95% CI = 0.32, 3.11; *P*‐value = 0.02) and giardiasis (Coefficient = 1.51; 95% CI = 0.79, 2.23; *P*‐value <0.001) were significantly associated with EED score. Children with EAEC had significantly higher MPO concentrations (Coefficient = 0.33; 95% CI = 0.06, 0.61; *P*‐value = 0.02).

**Conclusion:**

The study results imply the importance of intestinal pathogens in contributing to intestinal inflammation and increased intestinal permeability in young children.

## Abstract (fr)

**Objectif:**

La dysfonction environnementale entérique (DEE) peut être évaluée à l'aide de biomarqueurs fécaux tels que la myéloperoxydase (MPO), la néoptérine (NEO) et l'alpha‐1 anti‐trypsine (AAT). Nous avons cherché à tester l'association entre les pathogènes intestinaux et les marqueurs fécaux de la DEE chez les enfants vivant dans les bidonvilles au cours des deux premières années de la vie.

**Méthodes:**

Les données de la cohorte de naissance MAL‐ED du site du Bangladesh ont été utilisées pour effectuer cette analyse. Des analyses multivariées utilisant des équations d'estimation généralisées ont été effectuées pour tester l'association entre les pathogènes intestinaux et les marqueurs fécaux de la DEE.

**Résultats:**

La giardiase, l'ascaridiose et la trichocéphalose étaient les plus fréquentes infections parasitaires et *Campylobacter* spp., *Escherichia coli* entéroagrégative (EAEC) et *Escherichia coli* entérotoxigène (ETEC) étaient les agents pathogènes bactériens courants observés dans les échantillons de selles des enfants. Dans l'ensemble, 71%, 97% et 58% des échantillons de selles étaient au‐delà des valeurs considérées comme normales dans les milieux non tropicaux pour la MPO, la NEO et l'AAT, respectivement. La giardiase était significativement associée à la MPO (coefficient = 0,55; IC95%: 0,15 ‐ 0,95; valeur *p* = 0,008) et aux concentrations d'AAT (coefficient = 0,34; IC95%: 0,04 ‐ 0,63; valeur *p* = 0,03). Une association significative a été trouvée entre le trichocéphalose et la NEO (coefficient = 0,90; IC95%: 0,19 ‐ 1,61; valeur *p*= 0,01). La trichocéphalose (coefficient = 1,71; IC95%: 0,32 ‐ 3,11; valeur *p* = 0,02) et la giardiase (coefficient = 1,51; IC95%: 0,79 ‐ 2,23; valeur *p* <0,001) étaient significativement associées au score de DEE. Les enfants avec EAEC avaient des concentrations de MPO significativement plus élevées (coefficient = 0,33; IC95%: 0,06 ‐ 0,61; valeur *p* = 0,02).

**Conclusion:**

Les résultats de l’étude impliquent l'importance des agents pathogènes intestinaux dans l'inflammation intestinale et l'augmentation de la perméabilité intestinale chez les jeunes enfants.

## Introduction

Environmental Enteric Dysfunction (EED) is a subclinical intestinal disorder highly prevalent in low and middle income countries 1, 2. The pathology is associated with poor sanitation along with microbial and parasitic contamination of food and water and implicated in poor linear growth, impaired cognitive function and attenuated response to oral vaccines 2, 3, 4, 5. Evidence suggests that EED is a neglected but significant contributor to linear growth failure in countries such as Bangladesh where the aetiologies are more complex due to multifaceted interaction between numerous factors 6, 7, 8. EED refers to gut inflammation, increased intestinal permeability and reduced absorptive capacity in the small intestine 4. These features are manifested by shortening of villi, crypt hyperplasia and infiltration of lymphocytes into the lamina propria in histopathology of biopsied specimens collected from the small intestine 9, 10. Intestinal biopsy is the gold standard to diagnose EED, but the procedure is invasive and not feasible to conduct upon children 7, 10. Therefore, panels of biomarkers have been proposed to assess EED including Myeloperoxidase (MPO), Neopterin (NEO) and Alpha‐1 anti‐trypsin (AAT) 11. MPO and NEO are the specific markers of intestinal inflammation and AAT reflects enteric protein loss which is secondary to EED 12, 13, 14. However, it has already been posited that MPO, NEO and AAT can predict gut inflammation and increased intestinal permeability caused by EED 10, 15, 16, 17.

EED is also attributable to the insults of environmental origin caused by infectious agents including intestinal bacteria and parasites 11, 18. Intestinal infections are known to cause disrupted absorptive and barrier function in small intestine and also play a potential role in the development of undernutrition, particularly in children 19, 20, 21, 22, 23, 24. Exposures to intestinal parasites such as *Cryptosporidium* spp., *Giardia intestinalis, Ascaris lumbricoides* and *Trichuris trichiura* are common in developing countries and affect the nutritional status of children 25. Studies conducted in different parts of the world have reported that giardiasis, trichuriasis, ascariasis and cryptosporidiosis were associated with poor linear growth in children 26, 27, 28, 29. Evidence also suggests that infection with enteric bacteria for instance *Campylobacter* spp., Enteroaggregative *Escherichia coli* (EAEC), Enterotoxigenic *Escherichia coli* (ETEC) and Enteropathogenic *Escherichia coli* (EPEC) are linked with malnutrition and impaired cognitive function in young children 23, 30, 31. Intestinal pathogens also interrupt gut function and induce inflammatory responses in small intestine which are indicative of EED 27, 32, 33. But the definite contribution of different intestinal pathogens on gut inflammation and increased intestinal permeability caused by EED is poorly understood. Therefore, we aimed to examine the association of intestinal pathogens with faecal markers of EED (MPO, NEO and AAT) among slum‐dwelling children in the first 2 years of their life in Bangladesh.

## Methods

### Study setting and population

The Malnutrition and Enteric Disease (MAL‐ED) study was a multi‐country research activity which followed children longitudinally from birth to 2 years of age in eight countries 34. MAL‐ED birth cohort data from Bangladesh site were used to conduct this analysis. The detailed methodology of MAL‐ED study has been published elsewhere 35. The study was conducted in Bauniabadh area of Mirpur, an urban settlement in Dhaka, Bangladesh. A total of 265 healthy newborns living in the study area were enroled in the study within the first 17 days of life between February 2010 and February 2012. Exclusion criteria for cohort recruitment were maternal age <16 years, not a singleton pregnancy, another child already enroled in the MAL‐ED study, severe disease requiring hospitalisation prior to recruitment, and severe acute or chronic conditions diagnosed by a physician (e.g. neonatal disease, renal disease, chronic heart failure, liver disease, cystic fibrosis, congenital conditions).

### Ethical statement

The study protocol was reviewed and approved by the Ethical Review Committee of the International Centre for Diarrhoeal Disease Research, Bangladesh (icddr,b). Informed written consent was obtained from the parents or legal guardians of the participants enroled in the study.

### Data collection

Assessment of socioeconomic and household information was done at enrolment. Enroled participants were visited by MAL‐ED field staff every other day. Field research assistants interviewed the parents or caregivers using a structured and pre‐tested questionnaire. Biological samples including non‐diarrhoeal stool and blood were collected at 7, 15 and 24 months of age. Non‐diarrhoeal stool samples were considered as the specimens, which were collected two or more days after an episode of diarrhoea. The stool samples were collected without fixative by trained field staff. Plasma was obtained via centrifugation of the blood at 3005 g for 10 min. Stool and blood samples were stored in a −70 °C freezer prior to analysis.

### Laboratory analyses

All laboratory analyses were performed in the laboratories at icddr,b in Dhaka, Bangladesh. Stool samples were assayed using commercially available Enzyme Linked Immunosorbent Assay (ELISA) kits for MPO (Alpco, Salem, New Hampshire), NEO (GenWay Biotech, San Diego, CA) and AAT (Biovendor, Chandler, NC), following the instructions of the manufacturers. *Campylobacter* infection, giardiasis and cryptosporidiosis were diagnosed in stool samples using commercial ELISA kits according to standard instructions given by the producers. EAEC, ETEC and EPEC were detected using real‐time PCR (qPCR) assay. Ascariasis and trichuriasis were detected using wet preparation stool microscopy. Plasma zinc was measured by Atomic Absorption Spectometry. Ferritin and soluble transferrin receptor (sTfR) levels were measured using Chemiluminescence Immunoassay (CLIA) and Immunoturbidimetry respectively.

### Statistical analysis

Frequency with proportion estimate was reported for categorical variables and median with inter‐quartile range (IQR) for asymmetric quantitative variables. Kruskal–Wallis test was applied for comparing non‐parametric variables over months or between the groups. Faecal marker concentrations were categorised based on the distribution of all measurements: low (in first quartile), medium (within the inter‐quartile range [IQR]) or high (in fourth quartile). At each time point, the composite EED score ranging from 0 to 10 was calculated from the three faecal markers following the method published previously 15. In this study, intestinal pathogens were the predictors and individual faecal marker or the composite EED score was considered as outcome. All three faecal markers were log‐transformed before analysis. The relationship between predictor variables and outcome was examined using GEE model. The family was Gaussian, link function was identity and correlation matrix was unstructured. Multicollinearity among independent variables in each model was checked. Initially, bivariate analysis was performed to identify the unadjusted effect of each predictor on outcome through individual GEE model. Covariates for multivariable models were selected if their association with the outcome have significance of <0.2 in bivariate analysis. Then multivariable analyses were performed using GEE to test the associations between individual faecal markers or EED score with intestinal pathogens after adjusting for potential confounders. A probability of <0.05 was considered statistically significant and the strength of association was determined by the coefficient values and their 95% confidence intervals (CIs). All analyses were performed using STATA version 13.0 IC (College Station, Texas).

## Results

Of the 265 participants, samples were available from 222 for analysis at 7 months of age. Gender was equally representative at all three time points. The prevalence of stunting was 20.9%, 42.3% and 48.5% at 7, 15 and 24 months of age respectively. The average number of family members in the households was >5 and approximately four shared a room at night. The demographic characteristics of the study participants are presented in Table 1.

**Table 1:** Descriptive characteristics of the participants at 7, 15 and 24 months of age

Variables	7 months	15 months	24 months
Sex (female), *n* (%)	113 (51%)	105 (51%)	98 (50%)
Family size, mean (SD)	5.22 (2.29)		
People sleeping per room, mean (SD)	3.67 (1.12)		
WAMI index, mean (SD)^a^	0.56 (0.12)		
LAZ score, mean (SD)	−1.29 (1.03)	−1.81 (0.96)	−2.03 (0.95)
Stunting, *n* (%)	45 (20.9%)	85 (42.3%)	96 (48.5%)
Anaemia, *n* (%)	111 (65.3%)	97 (63%)	63 (40.4%)
MPO (ng/ml), median (IQR)	7628.0 (3668.4–15928.9)	3156.2 (1193.5–5545.8)	2713.7 (1105.6–4758.4)
NEO (nmol/l), median (IQR)	1175.7 (628.7–2350.5)	1271.0 (453.7–2661.4)	382.8 (226.8–784.1)
AAT (mg/g), median (IQR)	0.40 (0.22–0.68)	0.30 (0.17–0.57)	0.30 (0.16–0.53)
Zinc (mmol/l), median (IQR)^b^	11.0 (9.9–12.2)	11.0 (10.1–12.1)	11.8 (10.7–13.0)
Haemoglobin (g/dl), median (IQR)^b^	10.7 (10.1–11.3)	10.4 (9.5–11.3)	11.3 (10.1–12.1)
Ferritin (μg/l), median (IQR)^b^	30.5 (16.1–49.8)	12.6 (6.4–22.0)	8.2 (4.8–14.4)
sTfR (μg/ml), median (IQR)^b^	5.2 (4.4–6.7)	7.0 (5.2–9.7)	6.8 (5.3–10.8)

a The WAMI score (ranging from 0 to 1) is a measure of household socioeconomic status including access to improved Water, sanitation and hygiene (WASH), Assets, Maternal education and Income 36.

b Normal ranges: Zinc, ≥9.9 mmol/l 37; Haemoglobin, ≥11 g/dl; Ferritin, >12 μg/l 37, 38; sTfR, <8.3 μg/ml 38.

### Prevalence of intestinal infections

**Figure 1:**
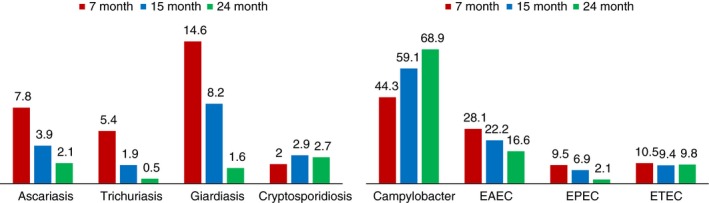
Prevalence of enteric infections at 7, 15 and 24 months of age. [Colour figure can be viewed at http://wileyonlinelibrary.com].

Giardiasis, ascariasis, trichuriasis and cryptosporidiosis were the most frequent parasitic infections in this cohort of children. The prevalence of all the parasitic infections by months of age is presented in Figure 1. The prevalence of ascariasis, trichuriasis and giardiasis were higher at 7 months of age and decreased with age (*P*‐value < 0.05). The prevalence of cryptosporidiosis was higher at 15 months followed by 24 and 7 months of age, although it was not statistically different (*P*‐value=0.82). Among the bacterial enteropathogens, *Campylobacter* spp., EAEC, ETEC and EPEC were observed most in the non‐diarrhoeal stool samples of the children (Figure 1). The prevalence of EAEC and EPEC infection fell significantly over the ensuing years (*P*‐value<0.05). In contrast, the prevalence of *Campylobacter* spp. in the non‐diarrhoeal stool samples of the children increased with age and that was statistically significant (*P*‐value < 0.001). The prevalence of ETEC infection was highest at 7 months of age, dropped at 15 months of age and remained almost the same at 24 months, (*P*‐value = 0.93).

### Faecal markers distribution in stool samples

**Figure 2:**
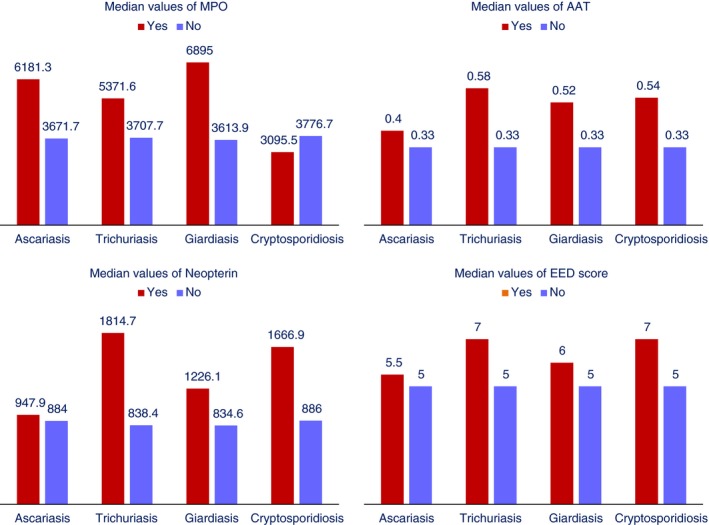
Overall median values of MPO, NEO, AAT and EED score in the stool samples with and without parasitic infections at 7, 15 and 24 months of age. [Colour figure can be viewed at http://wileyonlinelibrary.com].

There were 625 non‐diarrhoeal stool samples for which all three faecal marker test results were available. The overall median IQR values of MPO, NEO and AAT were 3895.4 (1565.2–8428.5) ng/ml, 890.8 (331.6–2089.0) nmol/L, and 0.33 (0.18–0.62) mg/g. The values observed in this cohort of children were considerably higher than non‐tropical standard values for the faecal markers. Overall, 71%, 97% and 58% of samples were above the values considered normal in non‐tropical settings for MPO, NEO and AAT [Non‐tropical standard: MPO < 2000 ng/ml, and NEO < 70 nmol/L, AAT < 0.27 mg/g 15]. Participants with parasitic infections had higher median values of MPO, NEO and AAT (Figure 2). Participants with ascariasis had higher median values of MPO (*P*‐value = 0.04) than normal children. Children with trichuriasis had higher median values of NEO (*P*‐value = 0.006) and AAT (*P*‐value = 0.03) than non‐infected participants. Those with giardiasis had higher median values of MPO (*P*‐value = 0.001) and AAT (*P*‐value = 0.04) relative to children without giardiasis. The median values of EED score were higher if the participants had trichuriasis (*P*‐value = 0.001) or giardiasis (*P*‐value = 0.004). The other differences in terms of median values were not statistically significant.

### Association of intestinal pathogens with faecal markers of EED and EED score

In multivariable analysis, giardiasis (Coefficient = 0.55; 95% CI = 0.15, 0.95; *P*‐value = 0.008) and EAEC infection (Coefficient = 0.33; 95% CI = 0.06, 0.61; *P*‐value = 0.02) were found to be significantly associated with faecal MPO concentrations after adjusting for sex, WAMI score, people sleeping per room, anaemia and ferritin. A significant association was observed between giardiasis and AAT concentrations (Coefficient = 0.34; 95% CI = 0.04, 0.63; *P*‐value = 0.03) after adjustment for sex and anaemia. Trichuriasis had significant association with faecal NEO (Coefficient = 0.90; 95% CI = 0.19, 1.61; *P*‐value = 0.01). The faecal markers were combined to form the composite EED score which was found to have association with trichuriasis (Coefficient = 1.71; 95% CI = 0.32, 3.11; *P*‐value = 0.02) and giardiasis (Coefficient = 1.51; 95% CI = 0.79, 2.23; *P*‐value < 0.001). However, the presence of *Campylobacter* spp., ETEC, EPEC, *Cryptosporidium* spp., and *A. lumbricoides* in non‐diarrhoeal stool samples were not associated with any of the faecal markers or EED score in the adjusted models (Table 2).

**Table 2:** Multivariable analyses for association of intestinal pathogens with faecal markers of EED and EED score using GEE

Variables	Unadjusted *β* (95% CI)	*P*‐value	Adjusted *β* (95% CI)	*P*‐value
Association of intestinal pathogens with Myeloperoxidase using GEE				
Sex (female)	0.20 (0.01–0.40)	0.04	0.17 (−0.06–0.40)	0.14
WAMI score	−0.67 (−1.48–0.14)	0.10	−0.24 (−1.22–0.75)	0.64
People sleeping per room	0.08 (−0.01–0.17)	0.07	0.07 (−0.03–0.18)	0.18
Ferritin	0.004 (0.001–0.006)	0.01	0.003 (0.001–0.006)	0.02
Anaemia	0.20 (−0.02–0.42)	0.08	0.17 (−0.07–0.40)	0.16
Ascariasis	0.64 (0.16–1.12)	0.01	0.32 (−0.17–0.82)	0.20
Trichuriasis	0.55 (−0.07–1.18)	0.08	0.60 (−0.15–1.35)	0.12
Giardiasis	0.59 (0.22–0.95)	0.002	0.55 (0.15–0.95)	0.008
EAEC	0.17 (−0.07–0.41)	0.16	0.33 (0.06–0.61)	0.02
ETEC	0.31 (−0.03–0.64)	0.08	0.13 (−0.23–0.49)	0.47
Association of intestinal pathogens with Neopterin using GEE				
Sex (female)	0.17 (−0.05–0.38)	0.14	0.15 (−0.07–0.38)	0.18
Trichuriasis	0.90 (0.19–1.62)	0.01	0.90 (0.19–1.61)	0.01
Association of intestinal pathogens with Alpha‐1 anti‐trypsin using GEE				
Sex (female)	0.08 (−0.08–0.23)	0.34	0.10 (−0.08–0.27)	0.28
Anemia	0.12 (−0.05–0.29)	0.17	0.09 (−0.08–0.27)	0.29
Trichuriasis	0.44 (−0.02–0.90)	0.06	0.31 (−0.24–0.87)	0.27
Giardiasis	0.29 (0.02−0.56)	0.03	0.34 (0.04–0.63)	0.03
Association of intestinal pathogens with EED score using GEE				
Sex (female)	0.49 (0.12–0.86)	0.01	0.52 (0.06–0.97)	0.03
Anaemia	0.42 (−0.004–0.84)	0.05	0.41 (−0.02–0.83)	0.06
Ferritin	0.01 (0.003–0.013)	0.001	0.01 (0.004–0.014)	<0.001
Trichuriasis	1.73 (0.57–2.89)	0.004	1.71 (0.32–3.11)	0.02
Giardiasis	1.25 (0.59–1.92)	<0.001	1.51 (0.79–2.23)	<0.001

## Discussion

This prospective longitudinal study found that giardiasis was significantly associated with faecal MPO and AAT concentrations of children under 2 years. *Giardia* is the commonest protozoan to cause intestinal parasitic infection during the first 2 years of life and is known for its deleterious effects on child growth 39. *Giardia* is also reported to be responsible for alteration of normal villous architecture and for infiltration of inflammatory cells in the lamina propria which are characteristic of EED 40. Moreover, a study conducted in rural Bangladesh documented that increased prevalence of giardiasis was associated with higher intestinal permeability measured by dual sugar permeability test 41. A previous report from multisite MAL‐ED data showed that presence of enteropathogens in non‐diarrhoeal stool samples was associated with elevated faecal MPO and AAT, the markers of intestinal inflammation and abnormal gut permeability 27. Consistent with those findings; our study results corroborate that *Giardia* infestation contributes to intestinal inflammation and increased gut permeability caused by EED.

The presence of EAEC in stool samples was also associated with elevated faecal MPO concentrations in this cohort of children. MPO is a marker of neutrophil activity, which is released in response to inflammatory changes in the intestinal mucosa 16, 42. Higher level of MPO in stool samples is suggestive of intestinal inflammation 16. Therefore, the association of EAEC with increased faecal MPO levels is consistent with previous literature demonstrating an association between EAEC and intestinal inflammation 43, 44.

We have observed a significant association between trichuriasis and faecal NEO concentrations in this cohort of children. NEO is a derivative of human monocytes and macrophages which is released in response to Th1 immune activation 45, 46. Previous reports documented that *Trichuris muris*, a commonly used laboratory model for *T. trichiura*, is associated with Th1 immune response 47. A study conducted in Gambia revealed that children with EED had Th1 type of immune response, which supports the possible mechanism by which trichuriasis contributes to elevated NEO, a marker of EED 48.

In the present study, the composite EED score was associated with both giardiasis and trichuriasis. The EED score was made combining the poorly correlated faecal markers (MPO, NEO and AAT) to better predict the intestinal dysfunction caused by EED 15, 16. The association of this composite score with giardiasis and trichuriasis postulate the pathway through which intestinal parasitic infections results in EED among susceptible paediatric populations, particularly those who are living in slums.

The most important insight of our study is the extent to which intestinal infections remains an uncharted frontier for child health in Bangladesh. Previous studies conducted in Bangladesh reported the presence of intestinal pathogens more often in non‐diarrhoeal stools of children 49, 50. We also found the persistence of intestinal pathogens in non‐diarrhoeal surveillance stools of the children at 7, 15 and 24 months of age. We observed higher prevalences of EAEC, ETEC, EPEC, giardiasis, trichuriasis and ascariasis at 7 months of age, immediately after cessation of exclusive breast feeding and introduction of complementary feeding. Perhaps the foods given to the children are contaminated with soil and water transmitted pathogens and responsible for such higher rates of enteric infections at 7 months of age. The prevalence of EAEC, ETEC, EPEC, giardiasis, trichuriasis and ascariasis decreased with increase of age, though the cumulative toll of these infections has been substantial among the enroled children. The reduction of prevalence over the ensuing years can be explained by improvement of hygiene and sanitation behaviour, changes in exposure, acquired immunity or combination of all these factors 51, 52, 53. *Campylobacter* spp. was the most prevalent pathogen in the stool samples of the children and gradually increased with age. This is in accordance with previous reports and supports the preponderance of *Campylobacter* spp. in resource poor settings 54. However, cryptosporidiosis has not been evident to the same degree as of other pathogens in this cohort of children at 7, 15 and 24 months of age.

In accordance with data from previous reports, our findings also demonstrated that the faecal marker levels were highly elevated among the enroled children compared to populations in non‐tropical settings. Furthermore, faecal marker values were significantly higher among those with any of the parasitic infections. This result indicates the pervasiveness of intestinal parasitic infections resulting in increased gut permeability and inflammation among children during first 2 years of life.

This study has several limitations. First, we could not demonstrate the causality of the association between the intestinal pathogens and faecal markers of EED. Second, the study was conducted in a setting where the elevated level of EED biomarkers is very common. Comparison with children from a higher socioeconomic class could help better understand the impact of enteric infections on faecal markers of EED. Despite the limitations, the study was well designed and equipped with skilled staff, followed rigorous enrolment criteria, and used high‐quality laboratory facilities.

In conclusion, presence of enteric pathogens in non‐diarrhoeal stool samples was associated with faecal biomarkers of EED in this cohort of children. Giardiasis was associated with higher faecal MPO and AAT concentrations in the stool samples of the children. Trichuriasis was also found to be significantly associated with elevated faecal NEO values. Among the bacterial pathogens, EAEC infection had significant association with increased MPO levels in stool samples. These findings imply the importance of enteric infections in contributing to intestinal inflammation and increased intestinal permeability among children in the first 2 years of life. The study results also warrant development of feasible interventions to reduce paediatric exposures to intestinal pathogens during the critical periods of early childhood.
